# Tracing COVID-19 Source of Infection Among Health Personnel in a Pediatric Hospital

**DOI:** 10.3389/fped.2022.897113

**Published:** 2022-06-09

**Authors:** Daniela de la Rosa-Zamboni, Fernando Ortega-Riosvelasco, Nadia González-García, Ana Estela Gamiño-Arroyo, Guillermo Alejandro Espinosa-González, Juan Manuel Valladares-Wagner, Araceli Saldívar-Flores, Olivia Aguilar-Guzmán, Juan Carlos Sanchez-Pujol, Briseida López-Martínez, Mónica Villa-Guillén, Israel Parra-Ortega, Lourdes María del Carmen Jamaica-Balderas, Juan José Luis Sienra-Monge, Ana Carmen Guerrero-Díaz

**Affiliations:** ^1^Department of Comprehensive Patient Care, Hospital Infantil de México Federico Gómez, Mexico City, Mexico; ^2^Department of Epidemiology, Hospital Infantil de México Federico Gómez, Mexico City, Mexico; ^3^Department of Research, Hospital Infantil de México Federico Gómez, Mexico City, Mexico; ^4^Department of Traumatology and Orthopedics, Hospital Regional Reynosa Mexican Petroleum, Mexico City, Mexico; ^5^Department of Nursing, Hospital Infantil de México Federico Gómez, Mexico City, Mexico; ^6^Department of Teaching and Research, Laboratorios Ruiz, Mexico City, Mexico; ^7^Department of Medical Management, Hospital Infantil de México Federico Gómez, Mexico City, Mexico; ^8^Department of Clinical Laboratory, Hospital Infantil de México Federico Gómez, Mexico City, Mexico; ^9^Department of Pneumology, Hospital Infantil de México Federico Gómez, Mexico City, Mexico; ^10^Department of Outpatient Pediatric, Hospital Infantil de México Federico Gómez, Mexico City, Mexico

**Keywords:** health personnel, COVID-19, hospital transmission, community transmission, SARS-CoV-2

## Abstract

Health personnel (HP) have been universally recognized as especially susceptible to COVID-19. In Mexico, our home country, HP has one of the highest death rates from the disease. From the beginning of the SARS-CoV-2 pandemic, an office for initial attention for HP and a call center were established at a COVID-19 national reference pediatric hospital, aimed at early detection of COVID-19 cases and stopping local transmission. The detection and call center implementation and operation, and tracing methodology are described here. A total of 1,042 HP were evaluated, with 221 positive cases identified (7.7% of all HP currently working and 26% of the HP tested). Community contagion was most prevalent (46%), followed by other HP (27%), household (14%), and hospitalized patients (13%). Clusters and contact network analysis are discussed. This is one of the first reports that address the details of the implementation process of contact tracing in a pediatric hospital from the perspective of a hybrid hospital with COVID-19 and non-COVID-19 areas.

## Introduction

Since the beginning of the COVID-19 pandemic, health personnel (HP) have been universally recognized as especially susceptible (1–4), with infection risks higher than the general population (5, 6). This is especially true in Mexico, our home country, where HP has one of the highest infection and death rates from COVID-19 (7, 8).

The source of infection for HP can be either in-hospital (from other healthcare providers or patients) or from the household or community, which has been reported as the main source of transmission (9). Also, since the pandemic affects mainly adults, the role of children and hospitalized pediatric patients in the transmission of SARS-CoV-2 are yet to be fully understood (10). The purpose of this work is to analyze the patterns and risks of transmission of HP in a pediatric hospital setting and to describe a customizable HP evaluation and monitoring plan.

## Materials and Methods

This is a descriptive, prospective, and observational study, which was carried out at the Children’s Hospital of Mexico Federico Gómez (HIMFG). The HIMFG is a tertiary care hospital with 290 beds, as well as an important teaching and research institute. At the beginning of the COVID-19 pandemic, the hospital was restructured to be able to have COVID areas as well as to trace both positive cases and contacts. Villa-Guillén et al. reported the restructuring of the hospital, in which it is mentioned that three COVID areas were established (11). The first area is for general pediatric care, the second for pediatric intensive care, and the third for neonatal intensive care. In addition, there is an area in the emergency room, which is intended for those patients with suspected COVID-19 who have not yet had an RT-PCR test. All areas had exclusive staff with clothing and washing areas. The use of personal protective equipment (PPE) is mandatory for all HP, ranging from a simple facemask for administrative staff to a three-layer mask, face shield, and/or glasses/goggles for clinical personnel. In-patient care, in the COVID-19 areas, requires the use of N95/KN95 respirators, goggles, gowns, double gloves, surgical suit, head cap, and boots, especially reinforced during aerosol-generating procedures.

### Health Personnel and Hospitalized Patients

By late March 2020, community transmission in Mexico was declared by the Ministry of Health (12), and a number of preventive measures were implemented at our hospital: (1) as of March 30, HP with comorbidities (non-compensated high blood pressure, uncontrolled diabetes, over 60-year-old, BMI >30, immune-compromised, etc.) were required to stay home; (2) as of April 21, universal masking of HP and face shields and/or glasses/goggles when having contact with patients was mandatory. All HP was trained on the use of PPE according to their specific tasks and work areas. Dining areas were furnished with acrylic divisions separating each seat, and hospital cafeterias served only take-out food. Lastly, to reduce the number of people and avoid crowding, HP was encouraged to work one-third of the time or fully telecommute if possible. Outpatient care was limited to emergencies, critical follow-ups, and non-deferrable interventions and surgeries; usual care for cancer, HIV, transplanted, and hemodialysis patients were not modified.

### Ambulatory Patients and Guardians

Checkpoints were implemented at the entrance of ambulatory patients and caretakers, to ensure proper use of facemask, check the temperature and respiratory symptoms, and supervised hand sanitation. Those patients or companions with respiratory symptoms were conducted through an outdoors cordoned route to a specific area for further clinical evaluation. Nasopharyngeal and pharyngeal RT-PCR for SARS-CoV-2 was performed in patients that fulfilled clinical criteria for COVID-19 assessment. By May, all patients scheduled for elective surgery were tested 24 h in advance for SARS-CoV-2 (by RT-PCR) before admission (emergency procedures patients were managed as COVID-19 positive). As of June, RT-PCR was performed on every patient 24 h prior to admission.

### Detection of Symptoms in Health Personnel

The department heads were given instructions, by the Epidemiology Department through a statement signed by the medical director, to notify all staff that if they presented symptoms they should go for a medical evaluation (including clinical evaluation, pulse-oximetry, and BMI determination) and assessment of co-morbidities. In addition to this, the indication was reinforced during educational sessions, daily rounds, and informative media. Daily rounds were conducted by designated HP from the Epidemiology Department for active surveillance of individuals with symptoms and referral for consultation if any. Causes of referral were: (1) presence of at least two of dysgeusia, arthralgia, myalgia, dyspnea, pharyngodynia, shivers, rhinorrhea, conjunctivitis, chest pain, or severe fatigue, and/or (2) at least one of cough, fever >38°C, dyspnea, or anosmia.

### Health Personnel Surveillance During the SARS-CoV-2 Outbreak With Ongoing Local Transmission

As of March 25 of 2020, all HP defined as “contacts” were clinically evaluated and tested (nasopharyngeal and pharyngeal RT-PCR). Contacts were defined according to the following criteria (13, 14): (a) proximity within 1.5 m for at least 15 min to a confirmed case, while both the case and the contact were not continuously wearing mouth, nose, and eye protection, (b) physical contact with a case without immediate hand hygiene, (c) contact with respiratory secretions, feces, and vomit without immediate hand hygiene or use of gloves, and (d) being in a room where aerosol-generating procedures were performed on a case (individual with an RT-PCR positive result) while not wearing an N-95 mask and goggles. These criteria had to be met during the period of maximum contagiousness, i.e., from 48 h before the case’s symptom onset and until 14 days afterward (13).

### Definition of Cases, Follow-Up, and Contact Tracing

All detected cases were instructed to isolate at home for 14 days and their clinical evolution was closely followed up during that period with periodical calls from an *ad hoc* Call Center, managed by the Hospital’s Epidemiology Department. For maximum protection for all HP patients and regardless of the RT-PCR result, HP was considered clinically positive if they presented fever >38°C, anosmia, or SpO2 <90% and were instructed to stay at home for 14 days.

During the first three months (until June 2020), all in-hospital contacts were temporarily removed from work, until the policy changed, and contacts could work if they remained asymptomatic.

The Call Center traced all in-hospital contacts for all cases; a pharyngeal and nasopharyngeal smear was taken for RT-PCR testing in all located contacts as symptoms developed, or 6–8 days after exposure to the confirmed case if asymptomatic. All these new contacts were followed up for 2 weeks and deemed cases of RT-PCR positive.

### Source of Infection and Contact Network

A possible source of infection was ascertained as (1) exposure to a confirmed hospitalized pediatric patient; (2) exposure to a suspected and/or confirmed COVID-19 case from their household; (3) exposure to another exposed HP during the contact tracing. Accordingly, cases were attributed to be acquired from (1) hospitalized patient, (2) household, (3) other HP, and (4) community/unknown (if no other clear source of infection was identified).

### Statistical Analysis

Parametric assumptions were adopted for descriptive statistics and non-parametric methods when the assumption of normality was not justified; covariates association was tested using logistic regression with the discretization of continuous and nominal covariates, and Chi-square was used to test the distribution of categorical variables. Degrees of contact were calculated using *epicontacts* (15).

### Ethics

In accordance with the Mexican Ministry of Health, health workers with symptoms should be tested for COVID-19 (16). Given that we followed this indication and the ones given by international guidelines (CDC) (17) no intervention was conducted, personal information was not included, and this was our standard of care, informed consent was not required. In addition to this, the Regulation of the General Health Law indicates in Article 17 that those studies in which no intervention is performed on the participants are considered studies without risk (18). However, this study is part of a larger study carried out within the HIMFG entitled “Risk factors for transmission and seroconversion of SARS-CoV-2 in HP. Cohort study in a Pediatric COVID Hospital” which was approved by both, the ethics committee and the research committee of HIMFG (approval number HIM-2020-031).

## Results

Of a total of 2,884 active hospital workers, 1,047 (36.3%) underwent clinical assessment and 850 (81.2%) required RT-PCR testing, of which 221 (26%) (7.7% of all personnel) were positive. A total of 28 (3.3%) personnel who underwent tests were non-conclusive; they were considered positive for personnel protection but were not integrated into the results of positive ones when doing the analysis comparing negative and positive cases. Table 1 shows the baseline characteristics of those who underwent clinical assessment.

**TABLE 1:** Baseline characteristics.

Characteristic	*n* = 1,047
Age (years) – mean (SD)	38.5 (10.1)
**Sex – no. (%)**	
Female	717 (68.5%)
Male	330 (31.5%)
Personnel working in other hospitals – no. (%)	78 (7.5%)
Personnel who live with someone who works in an other hospital – no. (%)	169 (16.1%)
Personnel who live with someone who works at the HIMFG – no. (%)	233 (22.3%)
**Comorbidities – no. (%)**	
Overweight	364 (34.8)
Obesity	285 (27.2)
High blood pressure	87 (8.3)
DM	36 (3.4)
COPD or asthma	55 (5.3)
Cardiovascular disease	10 (1)
Autoimmune disease	12 (1.1)
Smoking	68 (6.5)
Others	107 (10.2)
Any	745 (71.2)
**Job position – no. (%)**	
Nurses	402 (38.4)
Physicians	78 (7.4)
Medicine residents	230 (22)
Stretcher bearers and messengers	17 (1.6)
Cleaning and maintenance staff	70 (6.7)
Laboratory workers	32 (3.1)
Administrators	122 (11.7)
Dietitians and nutritionists	45 (4.3)
Security personnel	6 (0.6)
Inhalotherapists	13 (1.2)
Psychologists	17 (1.6)
Imageologists	5 (0.5)
Radiotherapists	2 (0.2)
Others	8 (0.8)

*SD, standard deviation; no., number; HIMFG, Hospital Infantil de México Federico Gómez; DM, diabetes mellitus; COPD, chronic obstructive pulmonary disease.*

Age, BMI, obesity, and other comorbidities were similarly distributed among all personnel studied, except for diabetes mellitus, 50% of the patients who had this disease were infected with COVID-19 (*p* = 0.007). Approximately 62% of all cases were overweight or obese. A total of 138 (13.2%) staff that underwent clinical assessment had at least one comorbidity, of which 86 (62%) (8.2% of all of the studied personnel) had overweight as the only comorbidity. The fact of working in the COVID-19 area was not associated with a positive test (*p* = 0.11). Being male and having diabetes mellitus increased the risk of positivity for SARS-CoV-2 (RR: 1.35 and 1.86, respectively). On the other side, medical residents and HP that lived with other people who worked in other hospitals reduced the risk (RR: 0.68 and 0.66, respectively). Baseline characteristics and PCR positivity can be seen in Table 2.

**TABLE 2:** Baseline characteristics and PCR positivity.

		Positive PCR	Negative PCR	
	Total	*n*	*n*	*p*
Sex – no. (%)	850	221	629	
Female	571	133 (60.2)	438 (69.6)	0.010
Male	279	88 (39.8)	191 (30.4)	
Personnel working in other hospitals – no. (%)	736	199	537	
Yes	71	14 (7.0)	57 (10.6)	0.144
No	665	185 (93.0)	480 (89.4)	
Personnel who live with someone who works in an other hospital – no. (%)	735	198	537	
Yes	153	30 (15.2)	123 (22.9)	0.022
No	582	168 (84.8)	414 (77.1)	
Personnel who live with someone who works at the HIMFG – no. (%)	734	198	536	
Yes	210	50 (25.3)	160 (29.9)	0.221
No	524	148 (74.7)	375 (70.0)	
Comorbidities– no. (%)	802	221	581	
Overweight	296	87 (39.4)	209 (36.0)	0.079
Obesity	220	59 (26.7)	161 (27.7)	0.737
High blood pressure	61	21 (9.5)	40 (6.9)	0.124
DM	24	12 (5.4)	12 (2.1)	0.007
COPD or asthma	43	11 (5.0)	32 (5.5)	0.938
Cardiovascular disease	9	1 (0.5)	8 (1.4)	0.304
Autoimmune disease	9	2 (0.9)	7 (1.2)	0.790
Smoking	61	11 (5.0)	50 (8.6)	0.137
Others	79	17 (7.7)	62 (10.7)	0.334
Job position – no. (%)	848	221	627	
Nurses	304	87 (39.4)	217 (34.6)	0.205
Physicians	76	16 (7.2)	60 (9.6)	0.297
Medicine residents	212	40 (18.1)	172 (27.4)	0.006
Stretcher bearers and messengers	15	6 (2.7)	9 (1.4)	0.215
Cleaning and maintenance staff	50	15 (6.8)	35 (5.6)	0.513
Laboratory workers	22	8 (3.6)	14 (2.2)	0.265
Administrators	90	25 (11.3)	65 (10.4)	0.695
Dietitians and nutritionists	36	11 (5.0)	25 (4.0)	0.530
Security personnel	6	3 (1.4)	3 (0.5)	0.382
Inhalotherapists	12	6 (2.7)	6 (1.0)	0.057
Psychologists	14	2 (0.9)	12 (1.9)	0.481
Imageologists	5	1 (0.5)	4 (0.6)	0.842
Radiotherapists	1	1 (0.5)	0 (0.0)	0.585
Others	5	0 (0.0)	5 (0.8)	0.412

*PCR, polymerase chain reaction; no., number; HIMFG, Hospital Infantil de México Federico Gómez; DM, diabetes mellitus; COPD, chronic obstructive pulmonary disease.*

Symptoms can be found in Table 3.

**TABLE 3:** Symptoms in positive and negative cases.

Symptoms	Negative PCR *n* = 629	Positive PCR *n* = 221	*p*
Fever – no. (%)	102 (16.2)	89 (40.3)	0.000
Cough – no. (%)	166 (26.4)	119 (53.8)	0.000
Headache – no. (%)	349 (55.5)	140 (63.3)	0.042
Dyspnea – no. (%)	58 (9.2)	44 (19.9)	0.000
Irritability – no. (%)	22 (3.5)	5 (2.3)	0.368
Desaturation – no. (%)	6 (1)	14 (6.3)	0.000
Diarrhea – no. (%)	109 (17.3)	28 (12.7)	0.105
Chest pain – no. (%)	73 (11.6)	47 (21.3)	0.000
Shivers – no. (%)	91 (14.5)	40 (18.1)	0.198
Pharyngeal pain – no. (%)	238 (37.8)	94 (42.5)	0.218
General discomfort – no. (%)	235 (37.4)	98 (44.3)	0.067
Rhinorrhea – no. (%)	131 (20.8)	68 (30.8)	0.003
Vomit – no. (%)	26 (4.1)	7 (3.2)	0.522
Arthralgias – no. (%)	159 (25.3)	70 (21.7)	0.065
Myalgia – no. (%)	208 (33.1)	113 (51.1)	0.000
Anosmia – no. (%)	17 (2.7)	29 (13.1)	0.000
Conjunctivitis – no. (%)	48 (7.6)	23 (10.4)	0.199
Nausea – no. (%)	17 (2.7)	4 (1.8)	0.617 (fisher)
Ear pain – no. (%)	6 (1)	0 (0)	0.348 (fisher)
Exanthema – no. (%)	13 (2.1)	0 (0)	0.026 (fisher)
Dysphonía – no. (%)	12 (1.9)	7 (3.2)	0.276
Abdominal pain – no. (%)	64 (10.2)	7 (3.2)	0.001
Dysgeusia/ageusia– no. (%)	10 (1.6)	14 (6.3)	0.000
Anorexia – no. (%)	2 (0.3)	3 (1.4)	0.114 (fisher)

*PCR, polymerase chain reaction; no., number.*

Healthy personnel with a positive test worked during the contagious period for a mean of 1.08 days (SD: 1.14) and were followed up for 14 days (SD: 14).

The most frequent symptoms were cough (53.67%) and myalgia (51.38%), followed by general malaise, pharyngodynia, and fever were present in about 40%, and chest pain in 21.1%. Finally, four patients (0.47% of people with a positive test) were hospitalized and two (0.24%) died.

Symptoms associated with a positive test were fever, cough, dyspnea, SpO2 <90%, chest pain, myalgia, dysgeusia, anosmia, rhinorrhea, and abdominal pain (*p* < 0.01).

### Contact Tracing

In-hospital tracing identified 517 HP contacts, of which 117 (22.6%) became confirmed cases. A median of zero SARS-CoV-2 contacts (IQR 0–0) was identified for each positive case (mean: 0.27, SD: 0.63). The HP who were infected from pediatric patients had attended a median of five patients with COVID-19 (IIC 1–10, min 1, max 124), during the 14 days prior to symptoms onset.

Out of the 221 HP that were RT-PCR positive, the source of infection could be identified as a hospitalized patient in 28 cases (12.8%), a household member in 30 (13.8%), a co-worker in 62 (28.4%), and community/unknown in 98 (44.9%).

**FIGURE 1:**
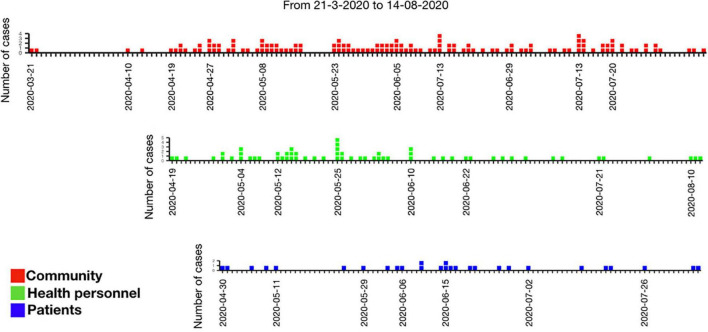
Number of cases according to the identified source (community, health personnel, or patients).

**FIGURE 2:**
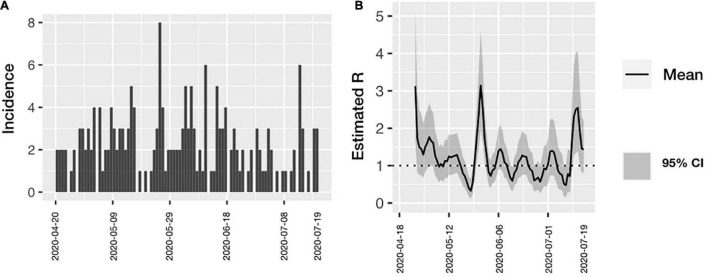
**(A)** Incidence of COVID-19 among all health personnel according with time of symptoms onset. **(B)** Estimated Rt over time in all health personnel. The effective transmission rate was calculated in all the hospital workers. Confidence Intervals are included.

Figure 1 shows the distribution of cases during the study period as well as the source of infection. A stable trend with occasional spikes can be observed. A gradual ascent was observed from May 22 of 2020 to May 25 when the maximum peak was reached; from then on and until June 10, cases were detected daily (Figure 1). At the end of June, the trend descended again and a further peak was observed on July the 13th. An Rt could not be calculated for individual hospital areas because of the scarcity of cases per area, although for the entire hospital Rt maximum of 3.2 to a minimum of 0.6 was calculated (Figure 2). The peaks occurred in mid and late May and early July. Both the higher incidence of cases and the increase in the effective transmission rate were associated with the dates when clusters occurred.

**FIGURE 3:**
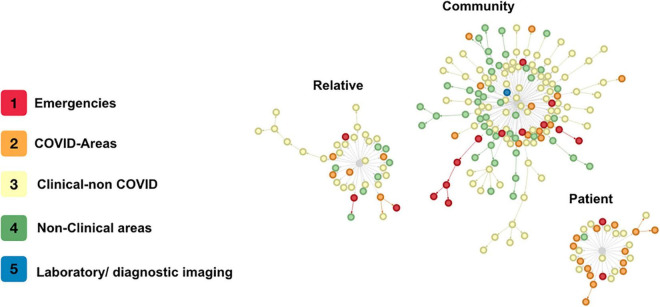
Contagion clusters. Every circle is a single health personnel with COVID-19. The colors indicate the area where each health personnel worked. The lines are the direction of contagion between contacts. The gray center is the type of source but not a single case of contagion.

A total of 13 clusters were identified (groups in which the unprotected case met the definition for “contact” simultaneously with several individuals, Figure 3). The cluster study required sampling 241 HP (23.9%), a median of 11 HP per cluster (IQR 7–17, min 5, max 90). In the clusters, 39 cases (17.9%) were identified, with a median of 2 secondary cases in each cluster (IQR 1–4.5, min 0, max 5) for each source case. In 8 of the 13 clusters studied (*N* = 128), the risk occurred in some type of meeting within the hospital.

## Discussion

Our protocol for the detection of symptomatic HP with COVID-19 and follow-up of contacts demonstrates that the main route of infection in HP in our hospital is their community, which was associated with longer and more complex chains of transmission, as evidenced in Figure 3. Hospital transmission dynamics showed a fluctuating effective transmission rate that rose significantly with meetings in closed spaces within the hospital. However, the average number of infections from each HP was <1, and transmission (except in a few cases) stopped before the third generation of cases. We found that most cases (44.9%) did not identify a household member or hospital coworker as potential sources of infection, and 28.4% had acquired it from a coworker; these findings have been previously described by other authors (2, 18).

Although community transmission as the highest transmission environment is not surprising (relaxation of personal protection behavior outside the hospital environment, a higher number of potentially infected contacts, etc.), coworker transmission as the second transmission environment deserves some consideration: it is possible that some HP may fail to identify themselves as contacts, leading to a careless behavior in the proper use of PPE (face mask, mainly), which could generate longer chains of contagions. On the other hand, awareness of being a contact may result in heightened self-protective behavior, limiting the chain of transmission.

The estimated Rt for the entire hospital doubled when calculated for transmission clusters in closed spaces. The transmission peaks within the hospital coincided with clusters detected where transmission was found mainly from other coworkers. It has been shown that the main risk factor for acquiring a respiratory disease in the hospital comes from a coworker and not hospitalized patients since coworkers usually interact for a longer time and at a shorter distance than they do with patients. Also, closed spaces are known to favor the transmission of SARS-CoV-2 (19). Park et al. in Korea found that the main route of transmission to HP in the hospital occurred in enclosed areas (20). Peaks of transmission rate over time coincide with greater community transmission in Mexico City, which suggests an important impact on transmission within hospitals, and the need for more rigorous in-hospital policies.

The average transmission for each infected individual using the proposed protocol was less than one, and transmission could be stopped in the second generation on average. Our study proposes a telephone follow-up and tracing strategy like suggested by the CDC (13), although, unlike the CDC approach, our HP with no symptoms continued working until their RT-PCR test results; this strategy did not impact the number of cases detected. With this protocol, it was possible to stop transmission at second generation or less in over 60% of all cases, with an average of 0 cases for each infected HP.

The percentage of HP diagnosed with COVID-19 in this study (7.7% of all HP currently working and 26% of the HP tested), was higher than in the general population, as previously described (1, 3–5) without this necessarily being explained by a higher exposure, but by epidemiological surveillance identifying subjects with minimal respiratory symptoms, and by less strict criteria used in the definition of the case and the purposeful focus on the contacts.

Our study has several limitations: first, it was carried out based on questioning the symptomatic HP and their coworkers, which could be subject to recall bias given the positive test result. However, statistical analysis was carried out taking into account only the temporal relation between cases, in which the highest transmissibility was determined according to the analysis and follow-up of contacts. On the other hand, the fact that it is a study carried out in a pediatric hospital may not make it generalizable to other types of adult hospitals, however, the average age range of the patients in the hospital has the same transmission risk as the adult population (10, 15). Also, the presence of SARS-CoV-2 in surfaces was not analyzed, nor was the effect of ventilation and crowding of each work area. However, the hospital reduced its bed and outpatient capacity to 50%, so crowding most likely was not a determinant factor in our results.

## Conclusion

In this study, we demonstrated that with proper use of in-hospital PPE, the main source of transmission for SARS-CoV-2 for HP was outside the hospital. Early detection of symptoms and contact screening are essential to halt internal transmission; it is not necessary to remove asymptomatic contacts from the work environment. It is imperative to develop public health and hospital policies focused on the promotion of personal protection measures in the community. It is urgent to develop guidelines to avoid transmission among coworkers, especially in closed, contained areas such as meeting rooms or dining rooms. Including personal hygiene, distancing, and ventilation requirements.

Having diabetes mellitus and being a man were associated with a higher percentage of positivity for COVID-19. However, these did not imply greater intra-hospital transmission. On the other hand, being a medical resident and living with someone that works at another hospital, was associated with a lower percentage of positivity.

Hospital workers are a very vulnerable resource in an epidemic and the implementation of protection measures and an active follow-up protocol with early detection of symptoms and contacts, effectively limits SARS-CoV-2 transmission to and from HP in the hospital, even without removing asymptomatic contacts from work. Most importantly, in a city with active transmission, the main route of infection in HP continues to be the community, so the continuation of in-hospital preventive measures is fully warranted outside the hospital environment. In addition, our findings can serve as an exhortation to develop community protection policies toward HP that limit their community exposure, as most surely COVID-19 will become an endemic condition even after the widespread application of the current vaccines.

## Data Availability Statement

The raw data supporting the conclusions of this article will be made available by the authors, without undue reservation.

## Ethics Statement

Ethical review, approval or written informed consent were not required for this specific study on human participants in accordance with the local legislation, as it is part of a larger study.

## Author Contributions

D-RZ contributed in the search for contacts, theoretical framework and literature search, data analysis, interpretation-analysis of results, discussion and conclusion, and manuscript preparation. FO-R contributed in the preparation of the database, search and follow-up of contacts, theoretical framework and literature search, data analysis, interpretation-analysis of results, discussion and conclusion, and preparation of the manuscript. NG-G contributed in the search for contacts and integration of contagion networks. AG-A contributed in the contact search, theoretical framework, and manuscript review. GE-G and JV-W contributed in the contact search, database filling for networks, and analysis of results. AS-F, OA-G, and JS-P contributed in the contact search and database filling for networks. BL-M, MV-G, and IP-O contributed with the interpretation of results. LJ-B helped coordination of personnel taking samples. JS-M coordinated the external consultation. AG-D contributed with the interpretation-analysis of results, discussion, and conclusion, and elaboration of the manuscript. All authors contributed to the article and approved the submitted version.

## Conflict of Interest

The authors declare that the research was conducted in the absence of any commercial or financial relationships that could be construed as a potential conflict of interest.

## Publisher’s Note

All claims expressed in this article are solely those of the authors and do not necessarily represent those of their affiliated organizations, or those of the publisher, the editors and the reviewers. Any product that may be evaluated in this article, or claim that may be made by its manufacturer, is not guaranteed or endorsed by the publisher.
